# Morbid obesity in women on the rise: an observational, population-based study

**DOI:** 10.1186/1471-2458-13-290

**Published:** 2013-04-02

**Authors:** Julie A Pasco, Sharon L Brennan, Mark A Kotowicz

**Affiliations:** 1 Epidemiology Unit for Musculoskeletal and Metabolic Disorders, School of Medicine, Deakin University, PO Box 281, Geelong, Victoria, 3220, Australia; 2 NorthWest Academic Centre, Department of Medicine, The University of Melbourne, St Albans, Victoria, Australia; 3 Department of Medicine, Barwon Health, Geelong, Victoria, Australia; 4 Australian Institute for Musculo-Skeletal Science, The University of Melbourne, St Albans, Victoria, Australia

**Keywords:** Morbid obesity, Women, Epidemiology, Body mass index

## Abstract

**Background:**

The obesity epidemic is generally monitored by the proportion of the population whose body mass index (BMI) exceeds 30 kg/m^2^ but this masks the growing proportion of those who are morbidly obese. This issue is important as the adverse health risks amplify as the level of obesity increases. The aim of this study was to determine how the prevalence of morbid obesity (BMI ≥ 40.0 kg/m^2^) has changed over a decade among women living in south-eastern Australia.

**Methods:**

BMI was determined for women in the Geelong Osteoporosis study (GOS) during two time periods, a decade apart. Height and weight were measured for 1,494 women (aged 20–94 years) during 1993–7 and for 1,076 women (aged 20–93 years), 2004–8, and the BMI calculated as weight in kilograms divided by the square of the height in metres (kg/m^2^). Prevalence estimates were age-standardised to enable direct comparisons.

**Results:**

Mean BMI increased from 26.0 kg/m^2^ (95%CI 25.7-26.3) in 1993–7, to 27.1 kg/m^2^ (95%CI 26.8-27.4) in 2004–8. During this period, the prevalence of morbid obesity increased from 2.5% to 4.2% and the standardised morbidity ratio for morbid obesity was 1.69 (95%CI 1.26-2.27). Increases in mean BMI and prevalence of morbid obesity were observed for all ages and across the socioeconomic spectrum.

**Conclusions:**

These findings reveal that over a decade, there has been an increase in mean BMI among women residing in south-eastern Australia, resulting in a measurable increase in the prevalence of morbid obesity.

## Background

The obesity epidemic in Australia is generally monitored by the proportion of the population whose body mass index (BMI) exceeds 30 kg/m^2^[1]. However, what is not as evident is the growing proportion of those who are morbidly obese. This issue is important as adverse health risks and healthcare expenditure amplify as the level of obesity increases [2]. Obesity-related health expenditure in Australia exceeded $8 billion in 2008, which included expenditure associated with metabolic disease, cardiac disease and surgical complications [3], but it is unclear how much of this expenditure related to different levels of obesity. Service providers are increasingly having to invest in equipment designed to accommodate the morbidly obese. It seems that both direct and indirect costs will amplify as the prevalence of morbid obesity increases.

In Australia, few, if any, studies have described the pattern of morbid obesity in the population. Thus, we aimed to document changes in the prevalence of morbid obesity (BMI ≥ 40.0 kg/m^2^) that have occurred over a decade among women living in south-eastern Australia.

## Methods

### Subjects

The Geelong Osteoporosis Study (GOS) is a population-based cohort study of adults aged 20 years and over, randomly-selected from the Commonwealth electoral rolls for the Barwon Statistical Division in south-eastern Australia [4]. In Australia, the electoral roll is a register of all eligible voters and provides the most comprehensive available list of adult Australian citizens. At baseline, an age-stratified sample was recruited so that there were approximately 100 individuals for each 5-year age-group 20–24, 25–29, 30–34, 35–39, 40–44, 45–49, 50–54, 55–59, 60–64 and 65–69 years, and approximately 200 individuals for each of the age groups 70–79 years and 80 years and older. Baseline assessments for women enrolled in the GOS occurred during the period 1993–7 (1,494 recruited, 77% response) and again approximately a decade later, 2004–8 (882 of the eligible women were assessed at the 10-year follow-up with 82% response). A further sample of 194 women aged 20–29 years was also randomly generated using the same methods employed for the baseline recruitment (2005–8, 82% response) and included in this study. Thus, data from 1,076 women were included in analyses for the 2004–8 time period. Most of the cohort (99%) was Caucasian; details of the study have been provided elsewhere [4]. All participants gave written, informed consent. The Barwon Health Human Research Ethics Committee approved the study.

Weight was measured to the nearest 0.1 kg using electronic scales, standing height was measured to the nearest 0.001 m using a wall-mounted stadiometer and BMI expressed as weight/height^2^ (kg/m^2^). An adult with a BMI of 40.0 kg/m^2^ or over is described as being morbidly obese (class III obesity) but the term can also refer to those who have a BMI over 35 kg/m^2^ with obesity-related co-morbidities [5]. We identified morbid obesity using BMI criteria alone. Class II obesity corresponded to BMI 35.0-39.9 kg/m^2^, class I obesity to BMI 30.0-34.9 kg/m^2^, overweight to BMI 25.0-29.9 kg/m^2^, normal weight to BMI 18.5-24.9 kg/m^2^ and underweight to BMI <18.5 kg/m^2^[6]. Area-based socioeconomic status (SES) was determined using the Socio-Economic Index for Areas (SEIFA) values based on census data (for 1996 for 1993–7; and for 2006 for 2004–8) from the Australian Bureau of Statistics (ABS). SEIFA values were used to assign an Index of Relative Socioeconomic Disadvantage, which was categorised into quintiles according to cut-off points for the study region.

### Statistical analysis

In order to compare the prevalence of each BMI category in 1993–7 and 2004–8, both time period datasets were stratified into age-groups and age-standardised to the 2006 census population figures for the Barwon Statistical Division (ABS Catalogue No. 2001.0). The standardized morbidity ratio (SMR) for morbid obesity was calculated using the 2004–8 dataset as the standard.

The distribution of BMI was skewed, but was normalised by natural log-transformation. Differences in mean BMI between the two time periods were determined using linear regression models. The variable of interest was the period of assessment (1993–7 or 2004–8) and the models were adjusted for age and SES tested as a potential confounder. Differences in the likelihood of morbid obesity during the two time periods were determined using logistic regression models. The outcome was morbid obesity and the exposure variable of interest was the period of assessment; models were adjusted for age and SES tested as a confounder. This method enables a comparison of the likelihood of morbid obesity between women of the same age and SES in the different time periods. In the statistical models, interaction terms were tested as effect modifiers and retained in the model if p < 0.05. Statistical analyses were performed using Minitab (version 15; Minitab, State College, PA).

## Results

Subject characteristics are shown in Table 1. The calculated age-adjusted geometric mean BMI increased from 26.0 kg/m^2^ (95%CI 25.7-26.3) in 1993–7, to 27.1 kg/m^2^ (95%CI 26.8-27.4) in 2004–8. The increase was observed across the age range, as evidenced by a non-significant age*time-period interaction in the regression model. Similarly, no interaction was detected between SES and the time period, indicating that mean BMI increased consistently across the range of SES.

**Table 1:** Subject characteristics for the two periods of assessment, 1993–7 and 2004–8

	**1993-7**	**2004-8**
	**n = 1,494**	**n = 1,076**
Age (years); median (interquartile range)	54.2 (37.4-71.6)	51.1 (34.7-65.9)
BMI categories*; n (%)		
morbid (class III) obesity	34 (2.3%)	44 (4.1%)
class II obesity	75 (5.0%)	81 (7.5%)
class I obesity	227 (15.2%)	191 (17.8%)
overweight	494 (33.1%)	333 (31.0%)
normal weight	635 (42.5%)	412 (38.3%)
underweight	29 (1.9%)	15 (1.4%)

*BMI (kg/m^2^) for morbid (class III) obesity ≥40.0, class II obesity 35.0-39.9, class I obesity 30.0-34.9, overweight 25.0-29.9, normal weight 18.5-24.9, underweight <18.5.

For the period 1993–7, 34 (2.3%) women were identified as morbidly obese (class III obesity), 75 (5.0%) as class II obesity and 227 (15.2%) as class I obesity; there were 494 (33.1%) women classified as overweight; 635 (42.5%) as having normal weight and 29 (1.9%) as underweight (Table 1). A decade later, 2004–8, 44 (4.1%) women were morbidly obese (class III obesity), 81 (7.5%) were class II obesity, 191 (17.8%) were class I obesity, and 333 (31.0%) were overweight, 412 (38.3%) were of normal weight and 15 (1.4%) were underweight.

The odds for morbid obesity were 76% greater in 2004–8 compared to 1994–7 (OR = 1.76, 95%CI 1.12-2.78). This association was sustained after adjusting for SES (data not shown) and found to be consistent across SES categories.

**Figure 1:**
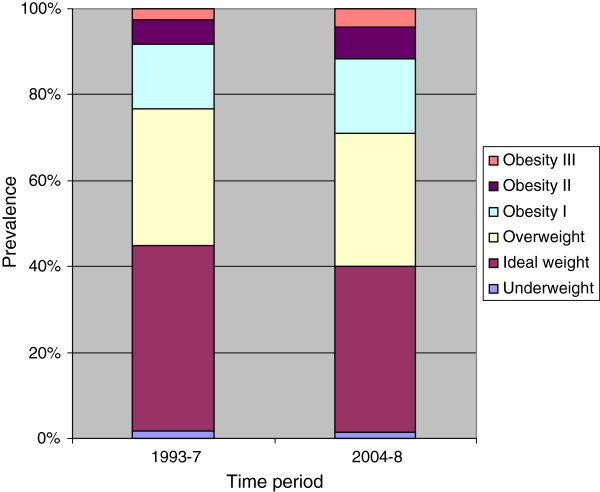
**Title: The prevalence of overweight and obesity according to BMI, for women residing in south-eastern Australia during 1993–7 and 2004–8.** Legend: The stacked column histogram displays the prevalence of morbid (class III) obesity (BMI ≥ 40.0 kg/m^2^), class II obesity (BMI 35.0-39.9 kg/m^2^), class I obesity (BMI 30.0-34.9 kg/m^2^), overweight (BMI 25.0-29.9 kg/m^2^), normal weight (BMI 18.5-24.9 kg/m^2^), and underweight (BMI < 18.5 kg/m^2^). Data are age-standardised to the 2006 census population figures for the Barwon Statistical Division (ABS Catalogue No. 2001.0) and presented for two time periods, 1993–7 and 2004–8.

The age-standardised prevalence for morbid obesity (class III obesity) was 2.5% for 1993–7 and 4.2% for 2004–7; prevalence figures for class II obesity were 5.6% and 7.3%, for class I obesity 15.1% and 17.6%, for overweight 31.8% and 30.8%, for normal weight 43.4% and 38.8%, and for underweight 1.6% and 1.3% (Figure 1). The SMR for morbid obesity (class III obesity) was 1.69 (95%CI 1.26-2.27).

## Discussion

We report that mean BMI has increased by 4.2% among women during the decade between 1993–7 and 2004–8. A comparison of the prevalence of morbid obesity in 2004–8 with 1993–7 generated a SMR for morbid obesity between 1.26 and 2.27, demonstrating a measurable increase over this ten year time period. Because of the approximate bell-shaped distribution of BMI in the population, a shift of the distribution to higher BMI resulted in a marked proportional increase in the prevalence of the more extreme BMI values. Disproportionate increases in the more extreme categories of BMI have been similarly reported for adults in the USA between 2000 and 2005 [7].

Our results indicate that the increase in the proportion of morbid obesity was not restricted to particular ages or different SES groups. Greater adiposity, and unhealthy lifestyle choices associated with increased risk for obesity, have been observed with increasing social disadvantage [8]. It is curious that our current study showed an increase in the prevalence of morbid obesity to have occurred across the SES spectrum. We may speculate that the increase in morbid obesity affects the population as a whole, thus shifting the entire population distribution toward greater risk for obesity-related disease. However, given the small number of morbidly obese women in our study, there may have been insufficient power to conclusively test for SES differences and it is plausible that a larger sample size could show inequity in the rise of morbid obesity between SES groups. Either way, our data suggest that population-wide approaches that change the underlying conditions in which behaviours occur could be warranted to combat rising rates of morbid obesity for all sub-groups of the population.

The strength of this study lies in the random nature of the study population and that the anthropometric measures used to calculate BMI were measured, rather than self-reported which would increase the potential for misclassification [9,10]. However, it is possible that participation bias may have changed over time. It is not unreasonable that social stigma linked with obesity [11] may change as the obesity epidemic progresses, and we cannot exclude the possibility that this might have differentially impacted on participation at the two time periods. Moreover, limitations of BMI as an indicator of adiposity have previously been demonstrated, especially for individuals with muscular body builds and the elderly [1], and this was not considered in the analyses.

Our study findings highlight the growing problem of morbid obesity associated with a population-wide increase in BMI. Recently, there has been increased advocacy in Australia for raising awareness of nutrition-related diseases and integrating nutrition into medical curricula [12]. In further recognition of issues surrounding the clinical management of morbidly obese patients, a Morbid Obesity model of care was developed in Western Australia in 2007 [13]. It should be clear, however, that unless the modern obesogenic environment is addressed, obesity-related health problems and healthcare costs are likely to escalate. In 2009, a parliamentary committee developed a series of recommendations designed to combat the rising rates of obesity in Australia [3]. Recommendations included development of the built environment to promote physically active lifestyles, education to encourage healthy eating and to increase the consumption of fresh fruit and vegetables, reformulation of commercially-available foods, more effective food labelling to assist healthy consumer choices and the development of a registry of bariatric surgery. While surgery and other gastrointestinal interventions may be considered appropriate for individuals with severe obesity and co-morbid disease, such as type 2 diabetes [5], such treatment requires a thorough multidisciplinary team assessment of peri-operative risk and possible long-term complications [14] and is unlikely to impact the rising prevalence of morbid obesity at a population level. The challenge is to identify and implement effective strategies that will shift the population distribution towards lower BMI, if the disproportionate increase observed in the extreme levels of obesity is to be curbed.

## Conclusions

We conclude that over a period of a decade, there has been an increase in mean BMI among women residing in south-eastern Australia, resulting in a measurable rise in the prevalence of morbid obesity.

## Abbreviations

ABS: Australian Bureau of Statistics; BMI: Body mass index; GOS: Geelong Osteoporosis Study; SEIFA: Socio-Economic Index for Areas; SES: Socioeconomic status; SMR: Standardized morbidity ratio

## Competing interests

No relevant disclosures.

## Authors’ contributions

JAP conceived of the study, developed the methods, conducted the literature search and led the development of the final manuscript. SLB and MAK contributed to the study conception, design and data interpretation, and provided intellectual content to the final manuscript. All authors read and approved the final manuscript.

## Pre-publication history

The pre-publication history for this paper can be accessed here:

http://www.biomedcentral.com/1471-2458/13/290/prepub
